# Long‐term persistence of Sox10CreERT2‐driven reporter labeling in enteric glia of mice under physiological and inflammatory conditions

**DOI:** 10.14814/phy2.71146

**Published:** 2026-11-03

**Authors:** Jacques Gonzales, Jonathon L. McClain, Brian D. Gulbransen

**Affiliations:** ^1^ Department of Physiology Michigan State University East Lansing Michigan USA; ^2^ Department of Physiology and Cell Biology University of Nevada Reno School of Medicine Reno Nevada USA

**Keywords:** Cre‐lox recombination, enteric glia, reporter, Sox10Cre^ERT2^

## Abstract

Inducible Cre‐lox systems are widely used to manipulate enteric glia in vivo. However, long‐term reporter expression following Cre‐mediated recombination remains poorly characterized, particularly under inflammatory conditions associated with cellular remodeling. This Methods Article evaluates reporter persistence within Sox10^+^ enteric glia over time. *Sox10* ^*CreERT2+/−*^ *; tdTomato* ^*fl/fl*^ reporter mice received two tamoxifen injections 48 h apart. Whole‐mount preparations of the longitudinal muscle‐myenteric and submucosal plexuses were analyzed by immunohistochemistry up to 7 months post‐induction. To assess tdTomato reporter expression persistence following inflammatory conditions, colitis was induced in mice after tamoxifen administration using dextran sulfate sodium (DSS) or dinitrobenzene sulfonic acid (DNBS), and reporter expression was quantified 1 month later. Tamoxifen treatment produced efficient tdTomato reporter labeling in Sox10^+^ enteric glia, with more than 95% of glial cells expressing tdTomato in both preparations. Reporter expression remained stable for up to 7 months under physiological conditions. Following DSS‐ or DNBS‐induced colitis, the proportion of tdTomato^+^/Sox10^+^ cells remained similar to healthy controls. Tamoxifen treatment induces efficient and durable reporter expression in Sox10^+^ enteric glia that persists for months under physiological conditions and up to 1 month following inflammation. This work establishes a practical framework for long‐term Sox10CreERT2‐driven reporter‐labeling studies of enteric glia.

## Graphical abstract

Sox10^CreERT2^‐driven tdTomato reporter labeling in enteric glia remained stable for up to 7 months under physiological conditions and was maintained for 1 month following DSS‐ or DNBS‐induced intestinal inflammation.

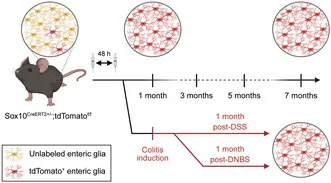

## 1 INTRODUCTION

The enteric nervous system (ENS) is an extensive network of neurons and glia within the walls of the intestine that provides moment‐to‐moment control of gastrointestinal motility, secretion, and barrier function (Sharkey & Mawe, 2023). Enteric glia are increasingly recognized as important regulators of gut allostasis, modifying signaling within neuronal circuits, epithelial integrity, and immune‐neural crosstalk, and altering ENS responses during disease states (Gonzales & Gulbransen, 2025). Inducible Cre‐lox genetic systems are widely used to study specific functions of enteric neurons and glia in vivo and enable temporal control of gene recombination in a defined cell population (Belkind‐Gerson et al., 2015; Joseph et al., 2011; Laddach et al., 2023; Laranjeira et al., 2011). Once induced, this strategy is generally assumed to provide durable expression in stable cell populations such as neurons; however, the long‐term stability of this approach in enteric glia, which can exhibit more plasticity and turnover in certain conditions, remains unclear. Thus, fundamental questions persist not only about enteric glial biology but also about the durability of genetic tools used to study them.

In other parts of the nervous system, glial populations can undergo proliferation, differentiation, or replacement in response to injury or disease (Bardehle et al., 2013; Stierli et al., 2018), suggesting that similar dynamics might occur in the gut. However, in the adult ENS, enteric glia appear largely stable during physiological conditions but display considerable plasticity during intestinal injury or inflammation (Belkind‐Gerson et al., 2015; Laddach et al., 2023; Laranjeira et al., 2011). Studies suggest that Sox10^+^ enteric glia contain a population of enteric neural progenitors capable of generating neurons following injury. These findings indicate that enteric glia can alter phenotype and lineage potential in response to environmental stimuli.

For many functional studies, enteric glia are genetically manipulated using inducible Cre‐lox systems for both reporter knock‐in and gene knockout strategies, most commonly via CreERT2 lines driven by glial genes such as *Sox10* or *Plp1* (Kabouridis et al., 2015; Rao et al., 2017; Woods et al., 2024). These approaches assume that, once induced, reporter expression persists within recombined cells and their progeny independently of subsequent CreERT2 or promoter activity. However, the long‐term persistence of reporter labeling in the ENS, particularly under conditions associated with cellular remodeling, has not been systematically tested. If newly generated cells arise from populations that escaped recombination at the time of tamoxifen administration, one would predict a progressive dilution of reporter‐positive cells over time. In contrast, if new glia arise from previously recombined Sox10^+^ cells or their progeny, lineage labeling would be expected to persist over time.

Here we employ an acute tamoxifen administration in a *Sox10* ^*CreERT2+/−*^ *; tdTomato* ^*fl/fl*^ reporter model to directly assess the long‐term persistence of Rosa26‐tdTomato reporter labeling in Sox10‐expressing enteric glia under physiological conditions and following inflammatory challenge.

## 2 MATERIALS AND METHODS

### 2.1 Animals

All animal procedures complied with the National Institutes of Health *Guide for the Care and Use of Laboratory Animals* and were approved by the Institutional Animal Care and Use Committee at Michigan State University (AUF# PROTO202400108). Mice were maintained under specific pathogen‐free conditions in facilities accredited by the Association for Assessment and Accreditation of Laboratory Animal Care (AAALAC) International. All experiments were performed using mice on a C57BL/6J background, housed in a temperature‐controlled environment on a 12‐h light/dark cycle (Optimice® cage system; Animal Care Systems, Centennial, CO) with ad libitum access to water and a minimal phytoestrogen diet (Envigo 2919, Indianapolis, IN). Sox10CreERT2 mice (MGI: 5301107), kindly provided by Dr. Vassilis Pachnis, were crossed with Rosa26‐CAG‐LSL‐tdTomato reporter mice (Ai14; Jackson Laboratory stock #007914).

Adult mice of both sexes (11 weeks old, *Sox10* ^*CreERT2+/−*^ *; tdTomato* ^*fl/fl*^ and *Sox10* ^*CreERT2−/−*^ *; tdTomato* ^*fl/fl*^) were administered tamoxifen (Sigma‐Aldrich T5648) dissolved in 90% ethanol and 10% sunflower oil at a dose of 10 mg/kg by intraperitoneal injection, twice at 48‐h intervals. This induction protocol was selected based on previous studies using the Sox10CreERT2 line (Laddach et al., 2023; Laranjeira et al., 2011; Morales‐Soto et al., 2024) and was found to produce efficient recombination in enteric glia. Intraperitoneal administration was preferred over a tamoxifen‐containing diet to ensure consistent dosing and to avoid potential confounding effects of prolonged tamoxifen exposure. Tamoxifen was administered 1 week prior to colitis induction to allow efficient reporter induction before the onset of inflammation, based on prior observations in this model.

For colitis induction, mice received either dextran sulfate sodium (DSS, 3% w/v in drinking water; MP Biomedicals 160110) for 7 consecutive days or dinitrobenzene sulfonic acid (DNBS, 0.2 mg/g body weight; Sigma‐Aldrich 556971) in 30% ethanol/70% saline (0.1 mL, intrarectal) under isoflurane anesthesia, beginning 1 week after tamoxifen induction. Two complementary models of colitis were used to capture distinct inflammatory contexts: DSS induces primarily mucosal inflammation, whereas DNBS produces primarily transmural inflammation. Body weight was monitored daily for 7 days following colitis induction.

### 2.2 Immunohistochemistry

Longitudinal muscle–myenteric plexus (LMMP) and submucosal plexus (SMP) whole‐mount preparations were obtained from mouse colon and fixed in 4% paraformaldehyde for 2 h at room temperature at 1, 3, 5, or 7 months post‐tamoxifen injection, or 1 month post‐colitis induction. Tissues were processed for whole‐mount immunohistochemistry as previously described (Morales‐Soto et al., 2023).

Briefly, LMMPs were rinsed three times (10 min each) in PBS containing 0.1% Triton X‐100, then incubated for 45 min in blocking solution of 4% normal donkey serum (Jackson ImmunoResearch, 017‐000‐121), 0.4% Triton X‐100 (Sigma, X100‐100 mL), and 1% bovine serum albumin (Jackson ImmunoResearch, 001‐000‐162). Preparations were incubated overnight at 4°C with primary antibodies: goat anti‐tdTomato (Sicgen AB8181, 1:500), rabbit anti‐Sox10 (Millipore HPA068898, 1:200), and mouse anti‐HuC/D (InVitrogen A21271, 1:200). After washing, tissues were incubated with Alexa Fluor–conjugated secondary antibodies anti‐goat 594 nm (Jackson ImmunoResearch, 705‐585‐147, 1:400), anti‐rabbit 647 nm (Jackson ImmunoResearch, 711‐605‐152, 1:400), and anti‐mouse 488 nm (Jackson ImmunoResearch, 715‐545‐150, 1:400) for 2 h at room temperature, then mounted in ProLong Diamond Antifade medium (Thermofisher P36984).

Fluorescence labeling was visualized using a Nikon Eclipse Ni upright epifluorescence microscope (20× objective, 0.75 NA, Plan Fluor) equipped with a Retiga 2000R camera (QImaging) controlled by Metamorph Software (Molecular device). Confocal representative images were acquired using a Nikon Ni‐E Microscope equipped with a Nikon AX Confocal system. Imaging was performed with the ApoLWD 25 × 1.10 WDIC N2 water immersion objective (numerical aperture, NA = 1.1), providing high resolution and optimal light collection for confocal imaging (2048 × 2048 resolution with line averaging of 2). Cell quantification was performed manually using Fiji (ImageJ). Quantification of positive Sox10^+^/tdTomato^+^ cells was performed on 5 images per animal in the longitudinal muscle‐myenteric plexus (LMMP) and at least 6 images per animal in the submucosal plexus (SMP). Ganglionic and extraganglionic Sox10^+^ glia were included in the myenteric plexus count, whereas muscularis‐associated glia adjacent to the myenteric plexus were quantified separately. Quantification of positive HuC/D^+^/tdTomato^+^ cells was performed on 5 images per animal in the LMMP preparations.

### 2.3 Statistical analyses

Data are presented as mean ± SD, with each data point representing one animal. Statistical comparisons were performed using GraphPad Prism. Group comparisons were performed using one‐way ANOVA followed by Bonferroni post hoc tests, with *p* < 0.05 considered statistically significant.

## 3 RESULTS

*tdTomato* expression was absent in *Sox10* ^*CreERT2−*^ controls, confirming the specificity of the reporter (Figure 1a,b). In *Sox10* ^*CreERT2+*^ animals not treated with tamoxifen, a small proportion of *tdTomato*‐positive cells was detected in both plexuses [myenteric plexus (MP): 23.6% ± 11.2%, submucosal plexus (SMP): 27.5% ± 8.1%] with no difference among sexes. All of these tdTomato‐positive cells co‐expressed Sox10, suggesting a low level of Cre‐leakiness in enteric glia in the absence of tamoxifen. In contrast, a higher proportion of tdTomato^+^/Sox10^+^ cells was observed in muscularis‐associated glia under this condition (42.1% ± 19.6%), indicating increased baseline recombination in this compartment.

**FIGURE 1:**
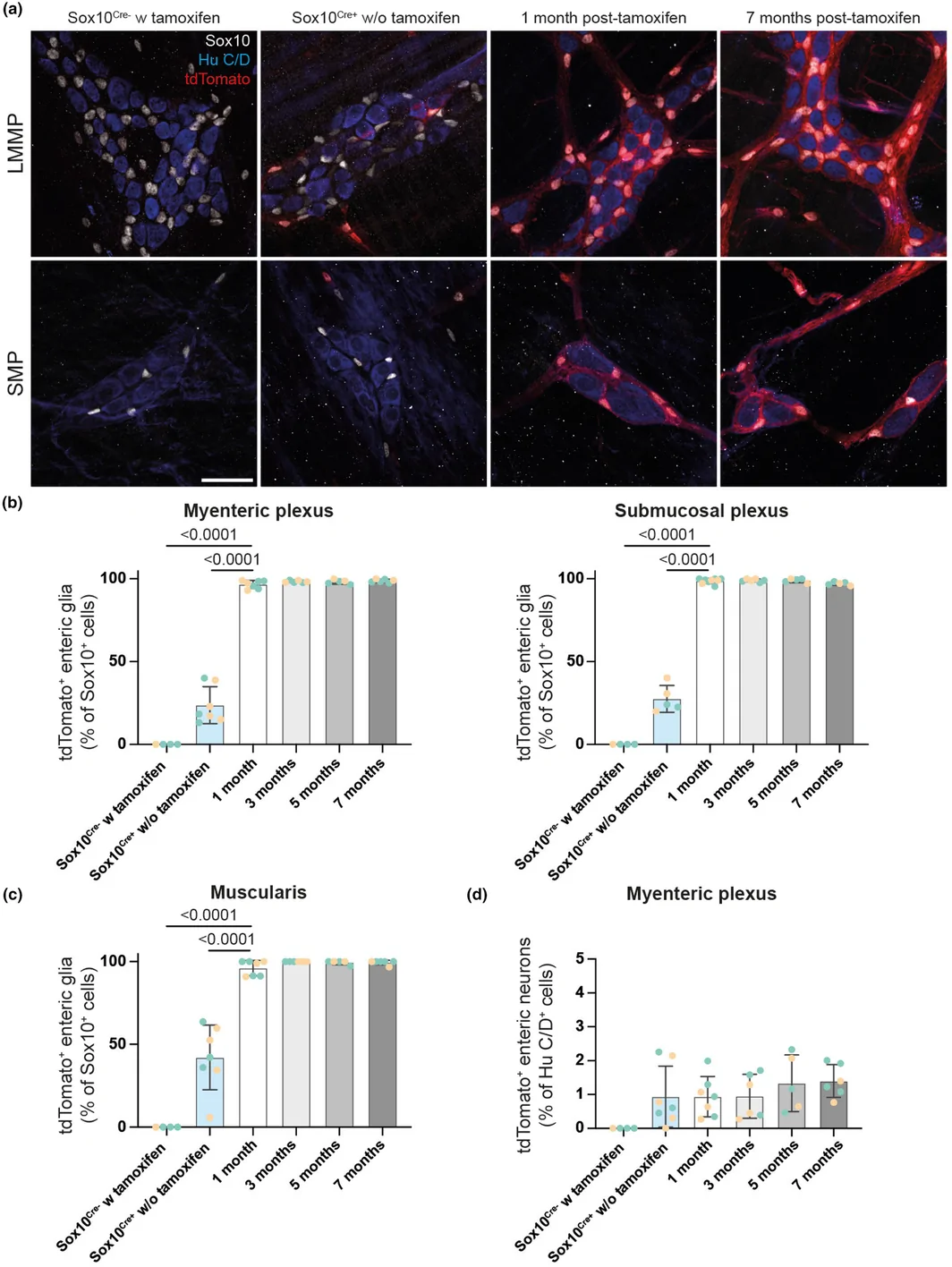
tdTomato^+^/Sox10^+^ cell quantification in the enteric nervous system following tamoxifen induction. (a) Representative images of tdTomato‐expressing Sox10^+^ cells in the myenteric plexus (LMMP, *top*) and submucosal plexus (SMP, *bottom*) from Sox10^Cre‐^ tamoxifen treated controls, Sox10^Cre+^ without tamoxifen and at 1, 3, 5 and 7 months following tamoxifen administration. tdTomato (red), Sox10 (white), HuC/D (blue). Scale bar, 50 μm. (b) Quantification of tdTomato^+^/Sox10^+^ cells in the myenteric and submucosal plexus. (c) Quantification of tdTomato^+^/Sox10^+^ cells in muscularis‐associated glia adjacent to the myenteric plexus. (d) Quantification of tdTomato^+^/HuC/D^+^ cells in the myenteric plexus. Each dot represents an individual animal (males, green; females, yellow). Data are presented as mean ± SD, *n* = 4–9 animals per group. Statistical analysis was performed using one‐way ANOVA with Bonferroni post hoc comparisons to the 1‐month group.

In contrast, following tamoxifen induction, nearly all Sox10^+^ glia in both the MP and the SMP robustly expressed tdTomato. In the MP, labeling reached 96.6% ± 2.4% at 1 month, 98.3% ± 0.6% at 3 months, 98.2% ± 1.3% at 5 months, and 98.8% ± 1.0% at 7 months post‐induction. In the SMP, it reached 98.7% ± 1.6%, 99% ± 0.9%, 99% ± 1.3%, and 97.1% ± 1.0% at the same time points, respectively. In muscularis‐associated glia, labeling reached 96.1% ± 4.6% at 1 month, 100% at 3 months, 99.4% ± 1.3% at 5 months, and 99.5% ± 1.3% at 7 months post‐induction (Figure 1c). Remarkably, this labeling remained stable for up to 7 months after induction. Notably, this long‐term stability is in line with previous observations that reported low enteric glial renewal in healthy adult mice, although without direct quantification (Joseph et al., 2011). Occasional *tdTomato*‐positive neurons were observed in LMMP preparations. Quantification revealed low and relatively stable proportions of tdTomato^+^/HuC/D^+^ neurons across times (0.94% ± 0.60% at 1 month, 0.95% ± 0.65% at 3 months, 1.33% ± 0.84% at 5 months, and 1.40% ± 0.48% at 7 months post induction; Figure 1d). These values are comparable to those observed in the absence of tamoxifen (0.93% ± 0.89%), suggesting that this low level of labeling may largely reflect baseline leakiness rather than ongoing glia‐to‐neurons conversion under steady‐state condition (Joseph et al., 2011).

To determine whether intestinal inflammation alters reporter expression in enteric glia, we examined *tdTomato* labeling 1 month after colitis induction using either dinitrobenzene sulfonic acid (DNBS) enema or dextran sulfate sodium (DSS, 3% for 7 days). This time point was selected to assess reporter persistence following acute inflammatory injury and early recovery. Previous studies have reported that inflammatory injury can remodel the ENS and have suggested proliferation and phenotypic changes within enteric glial populations (Belkind‐Gerson et al., 2015; Joseph et al., 2011). We therefore examined whether tdTomato reporter expression within Sox10^+^ enteric glia remained stable following inflammation.

In this context, we found that the proportion of tdTomato^+^/Sox10^+^ cells remained high 1 month after inflammation in both preparations (MP: DNBS 98% ± 1.3%, DSS 96.5% ± 2.0%; SMP: DNBS 97.9% ± 1.6%, DSS 97.8% ± 1.2%), values comparable to non‐inflamed controls (Figure 2a,b). Similarly, high levels of tdTomato^+^/Sox10^+^ labeling were maintained in muscularis‐associated glia (DNBS 99.3% ± 1.2%, DSS 98.9% ± 2.4%). The tdTomato labeling of Sox10^+^ enteric glia was maintained following acute inflammatory challenge. Notably, we observed an increase in tdTomato^+^/HuC/D^+^ neurons in the MP under inflammatory conditions compared with controls (control: 0.94% ± 0.60%; DNBS 1.86% ± 1.1%; DSS: 2.72% ± 1.2%; Figure 2d). Although this increase is consistent with previous reports suggesting glia‐derived neurogenesis during intestinal inflammation (Belkind‐Gerson et al., 2015; Joseph et al., 2011), this effect may also reflect, at least in part, Cre^ERT2^ leakiness or inflammation‐associated ectopic recombination.

**FIGURE 2:**
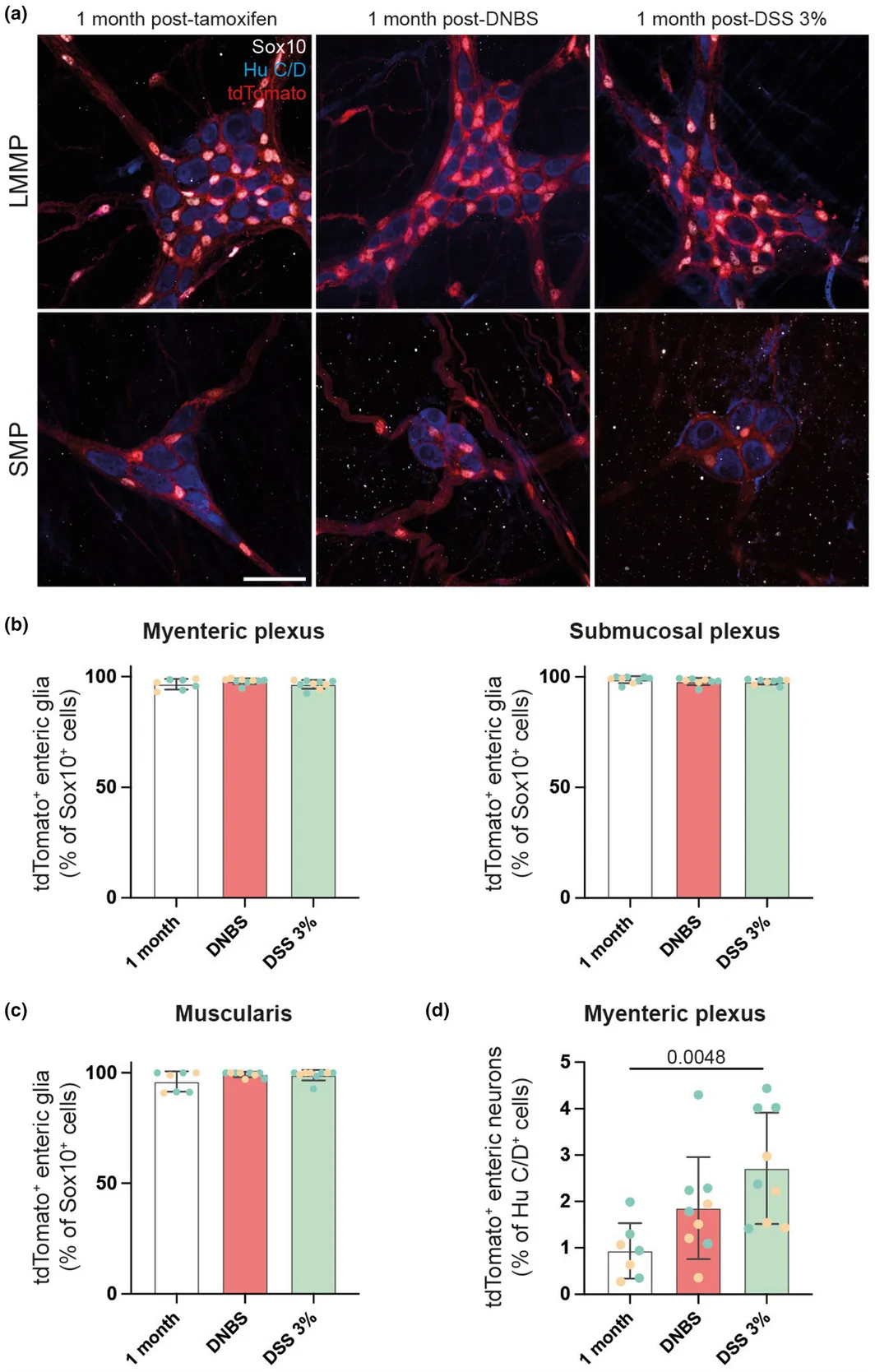
tdTomato^+^/Sox10^+^ cell quantification in the enteric nervous system following inflammatory colitis. (a) Representative images of tdTomato‐expressing Sox10^+^ cells in the myenteric plexus (top) and submucosal plexus (bottom) from healthy animals at 1 month post‐tamoxifen, and following DNBS‐ or DSS‐induced colitis. tdTomato (red), Sox10 (white), HuC/D (blue). Scale bar, 50 μm. (b) Quantification of tdTomato^+^/Sox10^+^ cells in the myenteric and submucosal plexus. (c) Quantification of tdTomato^+^/Sox10^+^ cells in muscularis‐associated glia adjacent to the myenteric plexus. (d) Quantification of tdTomato^+^/HuC/D^+^ cells in the myenteric plexus. Each dot represents one animal (males, green; females, yellow). Data are presented as mean ± SD, *n* = 7–9 animals per group. Statistical analysis was performed using one‐way ANOVA with Bonferroni post hoc comparisons to the 1‐month group.

## 4 DISCUSSION

Collectively, these observations demonstrate that an acute tamoxifen treatment of two injections separated by 48 h induces efficient and durable reporter expression following Cre‐mediated recombination in *Sox10* ^+^ enteric glia. The reporter expression persists for at least 7 months under physiological conditions and remains present 1 month after experimental colitis, with no evidence of significant reporter loss. To our knowledge, this is the longest follow‐up of Sox10^CreERT2^‐mediated reporter labeling in adult enteric glia and the first to describe the submucosal plexus, extending previous observations that followed labeled cells for approximately 2 months (Laranjeira et al., 2011).

These results directly address a crucial methodological concern: whether an acute tamoxifen administration is sufficient to maintain a high proportion of reporter‐labeled Sox10^+^ enteric glia over time, or whether repeated tamoxifen administration would be required to label newly generated glia coming from cells that escaped recombination at the time of initial induction. A proportion of tdTomato‐positive cells was observed in the absence of tamoxifen, indicating baseline Cre^ERT2^ leakiness. Ligand‐independent recombination has been widely reported in inducible Cre^ERT2^ systems, and the extent of such baseline recombination can vary across lines and is linked to promoter strength and construct design (Álvarez‐Aznar et al., 2020; Bedolla et al., 2024; Stifter & Greter, 2020). Importantly, recombination levels in the absence of tamoxifen remained substantially lower than those achieved following induction, supporting the robustness of the model for inducible targeting of enteric glia. Together, our data indicate that a short induction treatment is sufficient to maintain reporter expression over extended experimental time frames, thereby simplifying experimental design and reducing animal stress during long‐term studies.

These findings have several implications for studies of enteric glial biology. The maintenance of a high proportion of reporter‐labeled Sox10^+^ glia over time suggests that the proportion of reporter‐labeled cells remained stable during the time frame studied (up to 7 months following tamoxifen administration under physiological conditions). Cellular renewal would not necessarily dilute Sox10^+^ lineage labeling if newly generated cells derive from previously recombined cells or progenitors. In contrast, renewal from an unlabeled population could progressively dilute reporter labeling, as previously observed in a lineage‐tracing study of the adult ENS (Kulkarni et al., 2017). Therefore, our analysis does not exclude proliferation of already labeled glia or renewal from progenitors that had undergone recombination at the time of tamoxifen administration. Previous studies have shown that enteric glia can proliferate or generate neurons following inflammatory injury (Belkind‐Gerson et al., 2015; Woods et al., 2024), although our experimental approach does not directly measure glial proliferation or turnover.

We observed an increased proportion of tdTomato‐positive neurons after inflammation, with a significant increase following DSS‐induced colitis, while the increase observed with DNBS did not reach statistical significance. This finding is in line with previous reports suggesting that enteric glia can generate neurons following intestinal inflammation (Belkind‐Gerson et al., 2015; Joseph et al., 2011). However, this observation may also reflect low‐level Cre^ERT2^ leakiness or inflammation‐associated ectopic labeling. Therefore, this should be interpreted with caution. Taken together, these findings indicate that inducible reporter expression in Sox10^+^ cells appears to provide a stable readout of the labeled enteric glial compartment in both physiological and inflammatory states.

From a methodological standpoint, our study refines the use of the Sox10^CreERT2^ model in the enteric nervous system. Efficient reporter induction and sustained reporter expression can be achieved with two tamoxifen injections, avoiding the need for continuous dietary administration or repeated induction. Limiting prolonged tamoxifen exposure may reduce potential off‐target metabolic or hormonal effects and simplify experimental design, particularly in disease models where additional stressors are present. Moreover, the persistence of reporter expression in both the myenteric and submucosal plexuses supports the suitability of this approach for long‐term studies of enteric glia function in intestinal physiology and inflammation.

We acknowledge several limitations of this Methods Article. We did not directly assess cell proliferation using markers such as Ki67 or BrdU incorporation, and our analysis was confined to an acute colitis model. Moreover, proliferation markers may not fully capture enteric glial turnover, as prior studies suggest that under certain conditions, glia can differentiate into neurons (Laranjeira et al., 2011). In addition, we did not include mucosal enteric glia in our analysis. Although these cells represent a dynamic glial population, particularly in the context of inflammation, their reliable quantification using whole‐mount preparation remains technically challenging. An important consideration is that Sox10 is expressed in both mature enteric glia and progenitors. Therefore, stable reporter expression could reflect either long‐lived mature glia or renewal from pre‐recombined progenitor cells. Furthermore, reporter expression may depend on the reporter construct itself. A previous lineage‐tracing study in the ENS has reported cell‐type‐dependent differences in reporter expression and highlighted variability in CAG‐driven reporter expression (Kulkarni et al., 2023). Therefore, the stability observed in this study should be extrapolated with caution in the context of other reporter constructs. Also, whether similar stability would be observed using Cre drivers restricted to more differentiated glial populations, such as *Gfap*, *S100b*, or *Entpd2*, or under chronic inflammatory conditions remains to be determined. Such approaches may exclude progenitors and reveal different recombination dynamics over time.

In conclusion, we show that tamoxifen administration induces efficient and durable reporter expression in *Sox10* ^+^ enteric glia, that persists for months under physiological conditions and for 1 month following acute intestinal inflammation. These findings address a key technical question regarding the temporal stability of Sox10^CreERT2^‐based genetic strategies and support their suitability for long‐term reporter studies in the enteric nervous system. While our results do not address the extent of enteric glial turnover, they indicate that reporter expression following Sox10^CreERT2^‐mediated recombination provides a stable readout of the glial compartment over extended time frames. Together, this work establishes a practical framework for designing long‐term studies of enteric glial function and sets the stage for future investigations into glial plasticity, renewal, and aging within the enteric nervous system.

## AUTHOR CONTRIBUTIONS

**Jacques Gonzales:** Conceptualization; data curation; formal analysis; funding acquisition; investigation; methodology; resources. **Jonathon L. McClain:** Data curation; resources. **Brian D. Gulbransen:** Funding acquisition; supervision.

## FUNDING INFORMATION

J.G. receives support from the Research Fellowship Award (RFA) # 1160327 from the Crohn's & Colitis Foundation. B.D.G. receives support from grants R01DK103723 and R01DK120862 from the National Institute of Diabetes and Digestive and Kidney Diseases (NIDDK) of the National Institutes of Health (NIH). The content is solely the responsibility of the authors and does not necessarily represent the official views of the NIH.

## CONFLICT OF INTEREST STATEMENT

The authors declare no conflict of interest.

## ETHICS STATEMENT

All animal procedures were conducted in accordance with the National Institutes of Health Guide for the Care and Use of Laboratory Animals and were approved by the Institutional Animal Care and Use Committee at Michigan State University (AUF# PROTO202400108).

## Data Availability

Data are available from the corresponding author upon reasonable request.
